# Assessment of metabolic flux distribution in the thermophilic hydrogen producer *Caloramator celer* as affected by external pH and hydrogen partial pressure

**DOI:** 10.1186/1475-2859-13-48

**Published:** 2014-03-28

**Authors:** Alessandro Ciranna, Sudhanshu S Pawar, Ville Santala, Matti Karp, Ed WJ van Niel

**Affiliations:** 1 Department of Chemistry and Bioengineering, Tampere University of Technology, Korkeakoulunkatu 8, FI-33720 Tampere, Finland; 2 Department of Applied Microbiology, Lund University, P.O. Box 124, SE-221 00 Lund, Sweden

**Keywords:** *Caloramator*, Biohydrogen production, Metabolic flux analysis, Redox state, Hydrogen tolerance, Fermentation, Pyruvate node, Metabolic shift, Ethanol, Formate

## Abstract

**Background:**

*Caloramator celer* is a strict anaerobic, alkalitolerant, thermophilic bacterium capable of converting glucose to hydrogen (H_2_), carbon dioxide, acetate, ethanol and formate by a mixed acid fermentation. Depending on the growth conditions *C. celer* can produce H_2_ at high yields. For a biotechnological exploitation of this bacterium for H_2_ production it is crucial to understand the factors that regulate carbon and electron fluxes and therefore the final distribution of metabolites to channel the metabolic flux towards the desired product.

**Results:**

Combining experimental results from batch fermentations with genome analysis, reconstruction of central carbon metabolism and metabolic flux analysis (MFA), this study shed light on glucose catabolism of the thermophilic alkalitolerant bacterium *C. celer*. Two innate factors pertaining to culture conditions have been identified to significantly affect the metabolic flux distribution: culture pH and partial pressures of H_2_ (*P*_H2_). Overall, at alkaline to neutral pH the rate of biomass synthesis was maximized, whereas at acidic pH the lower growth rate and the less efficient biomass formation are accompanied with more efficient energy recovery from the substrate indicating high cell maintenance possibly to sustain intracellular pH homeostasis. Higher H_2_ yields were associated with fermentation at acidic pH as a consequence of the lower synthesis of other reduced by-products such as formate and ethanol. In contrast, *P*_H2_ did not affect the growth of *C. celer* on glucose. At high *P*_H2_ the cellular redox state was balanced by rerouting the flow of carbon and electrons to ethanol and formate production allowing unaltered glycolytic flux and growth rate, but resulting in a decreased H_2_ synthesis.

**Conclusion:**

*C. celer* possesses a flexible fermentative metabolism that allows redistribution of fluxes at key metabolic nodes to simultaneously control redox state and efficiently harvest energy from substrate even under unfavorable conditions (i.e. low pH and high *P*_H2_). With the H_2_ production in mind, acidic pH and low *P*_H2_ should be preferred for a high yield-oriented process, while a high productivity-oriented process can be achieved at alkaline pH and high *P*_H2_.

## Introduction

The need to circumvent environmental and social issues concerning the depletion of fossil fuels and the greenhouse gas emissions has led to exploration of alternative sources of energy. Hydrogen (H_2_) is considered as a promising energy carrier for the future because of its high energy content and non-polluting properties [1]. Moreover, it can be employed as a non-fuel commodity in a variety of industrial chemo-physical processes for which the demand is increasing [2].

Dark fermentation is a potential carbon neutral process for production of H_2_ from organic substrates by mesophilic or thermophilic anaerobic microorganisms. In order to establish an economically viable biological process for H_2_ production the yield needs to be maximized [3]. Mesophilic microorganisms are not capable of producing H_2_ at high yield being it reported in the range of 1-2 mol H_2_/mol hexose [4]. On the other hand, thermophiles have shown great potential for H_2_ generation, mainly because of the more favorable thermodynamics of the reaction at elevated temperatures which allows to generate a limited variety of by-products and to reach the theoretical yield of 4 mol H_2_/mol hexose [5].

*Caloramator celer*, formerly known as *Thermobrachium celere *[6], is a strict anaerobic, alkalitolerant, thermophilic bacterium capable of converting C_6_ sugars to H_2_, CO_2_, acetate, ethanol and formate as major metabolites. Previous studies have shown that *C. celer* produces H_2_ at high yields both in a naturally occurring microbial community and in pure culture [7-9]. However, the distribution of the end metabolites can vary depending on the growth conditions allowing the accumulation of significant amounts of ethanol and formate with consequent reduction of H_2_ yield.

Two innate factors pertaining to culture conditions have been identified to have a prominent role in the distribution of metabolic fluxes during anaerobic fermentation in *C. celer*: partial pressures of H_2_ (*P*_H2_) and culture pH [8,9]. The inhibitory effect on microbial growth and H_2_ production caused by H_2_ accumulation both in liquid and gaseous phases during the fermentation is a well known phenomenon and it has been demonstrated for several H_2_-producing organisms [9-13]. The fermentation medium can be easily supersaturated with H_2_ due to liquid-to-gas mass transfer limitations and this seems to be inevitable also at elevated temperatures [14,15]. In these conditions H_2_ synthesis becomes thermodynamically unfavorable and consequently the disposal of accumulated reducing equivalents in the cell is mediated by a metabolic shift towards production of more reduced metabolites, such as lactate, ethanol, acetone, butanol, or alanine [5]. As a consequence of the changes in the fermentation profile both H_2_ and ATP yields decrease. In previous studies *C. celer* achieved high H_2_ yields ( ≥ 3.3 mol H_2_/mol glucose) at low culture-to-headspace volume ratio and when H_2_ was periodically removed from the headspace [8,9]. These observations suggest that in order to maximize the H_2_ yield in *C. celer P*_H2_ in the fermentation vessel should be maintained at low level.

Culture pH is another factor affecting the distribution of metabolic fluxes during glucose fermentation. However, there exists a certain disagreement on the optimal pH to employ for H_2_ production [16]. Certainly, the optimal value needs to be studied case by case as it depends on the metabolic and physiological properties of the microorganism under investigation. Moreover, the ideal pH for cell growth may not be the same as the one for maximal H_2_ production [17].

Recently, a draft whole-genome sequence of *C. celer* has been obtained allowing to gain insight into the metabolic potential of this organism [18]. In particular, genomic analysis revealed pathways involved in pyruvate catabolism and end-product synthesis. To get a better understanding of the fermentative metabolism of *C. celer* under controlled conditions, a combination of experimental data from batch cultures in continuous stirred-tank reactor (CSTR), recently available genomic data and methods of metabolic flux analysis (MFA) were employed to analyze flux distribution towards end-products, taking in special consideration the effect of two innate factors, pH and *P*_H2_, on hydrogen production.

## Results

### Effect of culture pH on fermentative metabolism of *C. celer*

The effect of culture pH on growth and product formation in *C. celer* was investigated for the first time under pH-controlled conditions. Four different pH levels (8, 7, 6, 5.5) were tested during fermentation in a CSTR. At pH 8 and 7, *C. celer* showed about 3-fold higher maximum growth rates (*μ*_max_) than those obtained at pH 6 and 5.5 (Table 1). Similarly, higher glucose consumption rates were observed at pH 8 and 7 compared to pH 6 and 5.5. The highest biomass formation and biomass yield were similar at pH 8 and 7 and slightly lower at pH 6, while at pH 5.5 a significant reduction was observed (Table 1). Again, cultures at pH 8 and 7 displayed higher yield of biomass per ATP (Y_X/ATP_) than the one calculated at acidic pHs, whereas the ATP yield (Y_ATP/S_) increased at acidic conditions (Table 1).

**Table 1:** **pH-controlled batch fermentations of *C. celer *** **on 5 g/l of glucose at different culture pH**

		**pH**		
	**8**	**7**	**6**	**5.5**
*μ*_max_ (h^-1^)	1.50 ± 0.05	1.34 ± 0.05	0.53 ± 0.01	0.45 ± 0.06
Biomass concentration (g_CDW_/l)	0.95 ± 0.01	0.95 ± 0.06	0.76 ± 0.01	0.42 ± 0.01
*q*_glucose_^a^ (mmol/g_CDW_/h)	19.6 ± 1.4	20.6 ± 0.1	14.3 ± 0.1	14.0 ± 0.2
Y_X/ATP_ (g_CDW_/mol ATP)	15.4 ± 0.7	15.2 ± 0.4	6.8 ± 0.2	6.0 ± 0.4
Y_ATP/S_ (mol ATP/mol glucose)	2.81 ± 0.02	3.05 ± 0.03	3.54 ± 0.33	3.60 ± 0.09
H_2_ accumulation (mmol H_2_/l)	68.0 ± 1.3	64.2 ± 1.1	106.0 ± 2.3	91.7 ± 0.4
*Q*_H2_ (mmol H_2_/l/h)	25.2 ± 1.2	21.0 ± 1.3	14.9 ± 0.2	13.0 ± 0.9
Product yield (mol/mol glucose)				
Y_X_^b^	1.29 ± 0.05	1.37 ± 0.07	1.04 ± 0.01	0.62 ± 0.02
Y_H2_^b^	1.90 ± 0.01	1.69 ± 0.03	2.97 ± 0.10	2.48 ± 0.03
Y_A_^b^	1.04 ± 0.01	1.02 ± 0.04	1.33 ± 0.08	1.28 ± 0.04
Y_F_^b^	0.45 ± 0.01	0.37 ± 0.11	0.14 ± 0.02	0.19 ± 0.01
Y_E_^b^	0.35 ± 0.01	0.48 ± 0.05	0.20 ± 0.01	0.36 ± 0.03
RV_EP_^c^	2.60 ± 0.03	2.65 ± 0.13	3.36 ± 0.09	3.19 ± 0.02
HAc/EtOH	2.96 ± 0.11	2.15 ± 0.30	6.83 ± 0.67	3.60 ± 0.14
Carbon recovery (%)	88.6 ± 1.1	99.8 ± 1.5	100.7 ± 2.8	93.5 ± 1.6
Redox recovery (%)	94.4 ± 0.6	99.2 ± 0.7	98.4 ± 3.2	93.6 ± 2.1

^a^Specific glucose consumption rate measured during exponential phase.

^b^Y indicates the yield of product (where x=biomass, H_2_=hydrogen, A=acetate, F=formate and E=ethanol) per mole of glucose consumed.

^c^Total molar reduction values of reduced end-products (RV_EP =_ Y_H2_ + 2 * Y_E_) [19].

Besides growth, culture pH affected also the metabolite profile of *C. celer* during glucose fermentation. The highest H_2_ accumulation (106 mmol H_2_/l) and H_2_ yield (2.97 mol H_2_/mol glucose) were observed at pH 6, while at higher pH their values were about 33% lower (Table 1). In contrast, higher volumetric H_2_ productivity (*Q*_H2_) was obtained at alkaline conditions (25.2 mmol H_2_/l/h) and gradually decreased as the culture pH was reduced. The lower H_2_ production observed at pH 8 and 7 was accompanied by an increase of formate and ethanol yields, both being about 2-fold higher compared to those observed at pH 6 (Table 1). The lower RV_EP_ (total molar reduction values of reduced end-products) [19] and the acetate-to-ethanol ratio calculated for fermentation at pH 8 and 7 reflected the increased production of formate and ethanol (Table 1).

### Combined effect of culture pH and *P*_H2_ on fermentative metabolism of *C. celer*

#### *Combined effect of pH and P_H2_ on growth and metabolite production profiles*

**Figure 1:**
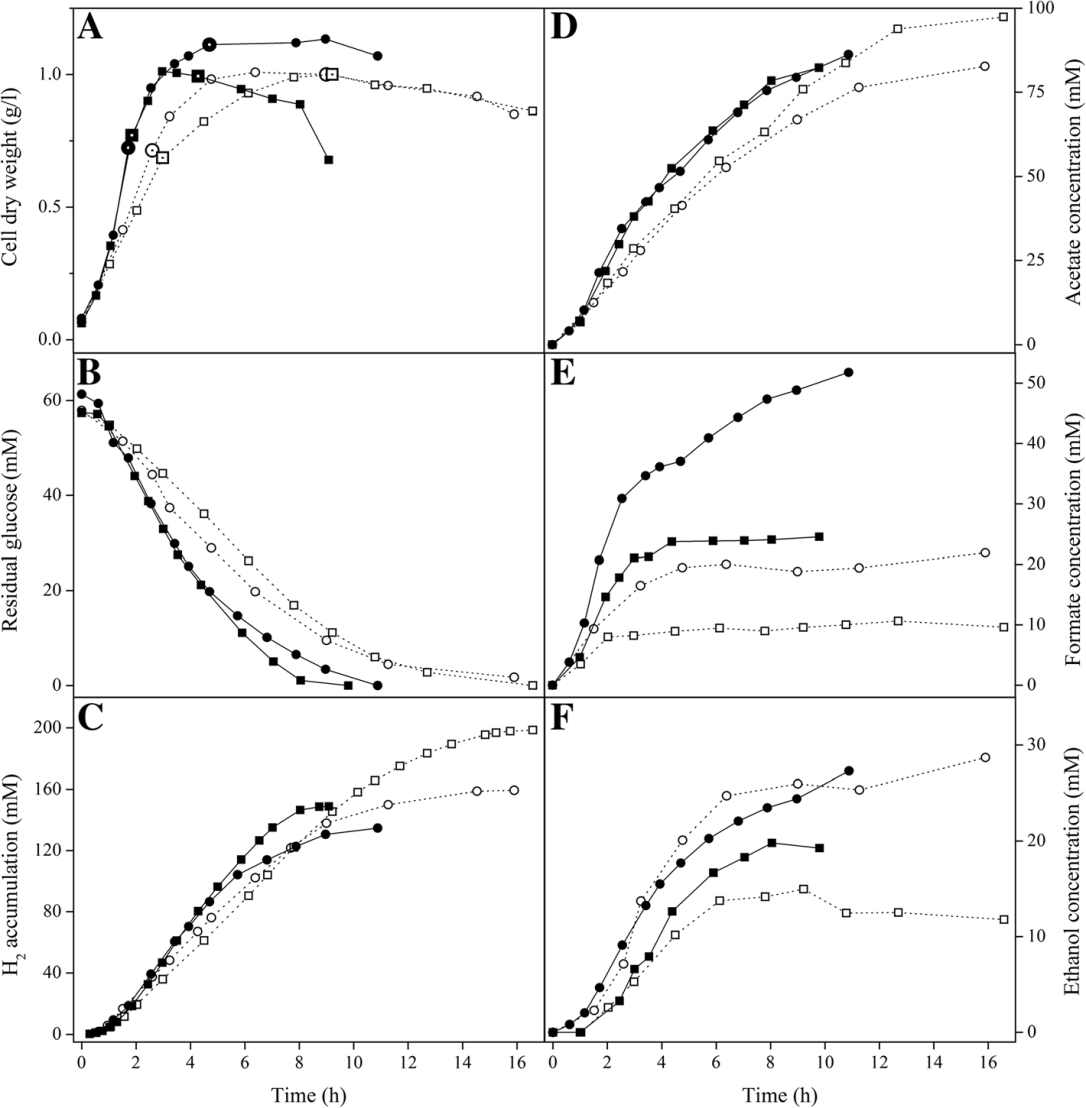
**Growth and product formation by *C. celer *in pH-controlled batch fermentations performed at pH 7 and 6, with and without N**_**2 **_**sparging.** Growth **(A)**, glucose consumption **(B)** and end product accumulation **(C-F)** profiles of *C. celer* cultured with 10 g/l glucose at pH 7 (*straight lines*) with N_2_ sparging (*Case I*, *filled squares*) and without N_2_ sparging (*Case II*, *filled circles*), and at pH 6 (*dashed lines*) with N_2_ sparging (*Case III*, *open squares*) and without N_2_ sparging (*Case IV*, *open circles*). Larger symbols in panel A represent sampling points for ATP, NADH and NAD^+^ measurements. Data are from one representative fermentation per condition.

To investigate the combined effect of pH and *P*_H2_ on growth, product formation and carbon flux distribution in *C. celer*, four different experimental conditions were tested using pH and application of N_2_ sparging as variables (*Case I*-*IV*). Sparging the reactor with N_2_ did not significantly affect the growth of *C. celer* at pH 7 and 6 (Figure 1A). In fact, despite the remarkable difference in the *P*_H2max_ in the reactor headspace with and without N_2_ sparging, similar growth rates and biomass yields were achieved within the same pH level (Table 2). On the other hand, at same sparging conditions the pH had a higher impact on growth with about 2.5-fold reduction of *μ*_max_ (Table 2) and a slower overall glucose consumption rate observed at pH 6 (Figure 1B). Also, Y_X/ATP_ was lower at pH 6, especially under sparging conditions (Table 2). Nevertheless, despite a slower growth at pH 6, the highest biomass formation was comparable to the one reached at pH 7 (about 1 g_CDW_/l) (Figure 1A).

**Table 2:** **pH-controlled batch fermentations of *C. celer *** **on 10 g/l of glucose at pH 7 and 6, with and without N**_**2 **_**sparging**

	**pH 7**		**pH 6**	
	**N**_**2 **_**sparging ( *Case I *)**	**No sparging ( *Case II *)**	**N**_**2 **_**sparging ( *Case III *)**	**No sparging ( *Case IV *)**
*P*_H2max_ (kPa)	8	69	6	74
*μ*_max_ (h^-1^)	1.09 ± 0.09	1.09 ± 0.08	0.43 ± 0.03	0.38 ± 0.05
Biomass concentration (g/l)	0.99 ± 0.03	1.13 ± 0.01	1.02 ± 0.03	1.01 ± 0.01
Y_X/ATP_ (g_CDW_/mol ATP)	11.5 ± 0.3	9.3 ± 0.1	6.3 ± 0.4	7.9 ± 0.2
Y_ATP/S_ (mol ATP/mol glucose)	3.24 ± 0.03	3.18 ± 0.02	3.4 ± 0.08	3.48 ± 0.17
*Q*_H2_ (mmol H_2_/l/h)	27.1 ± 1.5	33.0 ± 0.1	19.9 ± 0.8	20.6 ± 0.5
Product yield (mol/mol glucose)				
Y_X_^a^	0.71 ± 0.01	0.75 ± 0.03	0.72 ± 0.01	0.75 ± 0.01
Y_H2_^a^	2.32 ± 0.13	2.06 ± 0.13	3.10 ± 0.06	2.60 ± 0.03
Y_A_^a^	1.24 ± 0.01	1.28 ± 0.01	1.47 ± 0.07	1.35 ± 0.02
Y_F_^a^	0.37 ± 0.02	0.71 ± 0.12	0.14 ± 0.07	0.33 ± 0.06
Y_E_^a^	0.27 ± 0.01	0.37 ± 0.01	0.10 ± 0.01	0.39 ± 0.03
RV_EP_^b^	2.87 ± 0.11	2.8 ± 0.12	3.31 ± 0.08	3.39 ± 0.03
HAc/EtOH	4.5 ± 0.2	3.5 ± 0.1	14.2 ± 1.0	3.4 ± 0.3
Carbon recovery (%)	92.6 ± 0.6	94.0 ± 0.7	94.8 ± 4.2	100.3 ± 1.9
Redox recovery (%)	90.0 ± 0.6	97.5 ± 0.6	93.2 ± 4.1	102.3 ± 2.2

^a^Y indicates the yield of product (where x=biomass, H_2_=hydrogen, A=acetate, F=formate and E=ethanol) per mole of glucose consumed.

^b^Total molar reduction values of reduced end-products (RV_EP =_ Y_H2_ + 2 * Y_E_) [19].

Both culture pH and *P*_H2_ influenced the carbon flux distribution and the end-product profile in *C. celer* during glucose fermentation. The beneficial effect of H_2_ removal from the reactor on H_2_ production was more evident at pH 6 (*Case III* and *IV*). In fact, at this pH, when N_2_ sparging was applied, H_2_ accumulation increased by about 26% and H_2_ yield by 19% (*Case III*) (Figure 1C; Table 2). On the other hand, at pH 7 (*Case I* and *II*) the effect of *P*_H2_ on H_2_ production was marginal with a 9% and 13% increase at low *P*_H2_ for H_2_ accumulation and H_2_ yield, respectively (*Case I*). In addition, applying N_2_ sparging did not affect *Q*_H2_ within the same pH level. At the same sparging condition acidic pH promoted higher H_2_ accumulation and H_2_ yield, while neutral pH favored higher *Q*_H2_ (Figure 1C; Table 2). In fact, when N_2_ sparging was applied (*Case I* and *III*) H_2_ accumulation increased by 32% and H_2_ yield by 33% as pH set-point was lowered from 7 to 6. Even so, the *Q*_H2_ at pH 7 was about 36% higher than at pH 6. A similar trend could be observed without N_2_ sparging (*Case II* and *IV*), although the differences between H_2_ accumulation and H_2_ yield at pH 6 and pH 7 were significant but less pronounced.

Carbon and electron flow at the pyruvate and acetyl-CoA nodes was affected by both pH and *P*_H2_. While acetate accumulation profile and yield were minimally influenced by the *P*_H2_, formate and ethanol synthesis significantly increased at both pH levels when H_2_ concentration was allowed to build up in the reactor (*Case II* and *IV*) (Figure 1D, 1E, 1F; Table 2). Specifically, formate accumulation and yield were about 2-fold higher without N_2_ sparging regardless of the culture pH (*Case II* and *IV*), whereas the effect of *P*_H2_ on ethanol production was more drastic at pH 6 (*Case IV*) with an increase of accumulation and yield of 2.5- and 4-fold, respectively. The ability of *C. celer* to produce reduced end-products (RV_EP_), i.e. H_2_ and ethanol, remained unchanged under different *P*_H2_ (Table 2). On the other hand, acetate-to-ethanol ratio showed that at the acetyl-CoA node the redirection of carbon and electron flow toward ethanol synthesis is less favorable at low *P*_H2*,*_ especially at pH 6 (*Case III*) where the highest acetate-to-ethanol ratio of 14.2 was observed (Table 2).

Acidic pH (*Case III* and *IV*) promoted a slight increase of acetate accumulation and yield, whereas formate accumulation and yield were reduced by more than 2-fold at pH 6 both with and without gas stripping (*Case III* and *IV*) (Figure 1D, 1E; Table 2). A drastic reduction of ethanol synthesis occurred only at acidic pH with N_2_ sparging (*Case III*), while at high *P*_H2_ both ethanol accumulation and yield remained at high level regardless of the culture pH (*Case II* and *IV*) (Figure 1F; Table 2). The increase of RV_EP_ from 2.8-2.9 at pH 7 (*Case I* and *II*) to 3.3-3.4 at pH 6 (*Case III* and *IV*) indicates that pH influenced the conversion of glucose to reduced end-products (H_2_ and ethanol). The flux at the acetyl-CoA node was affected by pH only when sparging was applied resulting in a 3-fold increase of the acetate-to-ethanol ratio at pH 6 (*Case III*), while no variation in the ratio was observed when H_2_ concentration was allowed to build up in the reactor.

#### *Combined effect of pH and P_H2_ on ATP and redox levels*

To evaluate the effect of pH and *P*_H2_ on energy and redox metabolism of *C. celer*, intracellular ATP, NADH and NAD^+^ were measured in the exponential and stationary phase of growth (Figure 1A). At the same culture pH ATP levels were not significantly different both in exponential and stationary phase regardless of the application of N_2_ sparging, suggesting that intracellular ATP was not affected by the *P*_H2_ (Table 3). In contrast, culture pH showed a more significant influence on the ATP levels. In the exponential phase ATP was at least 2-fold higher at pH 7 (*Case I* and *II*) compared to pH 6 (*Case III* and *IV*). In the stationary phase intracellular ATP at pH 7 doubled (*Case I* and *II*), while at pH 6 was reduced by one-third (*Case III* and *IV*). As a consequence the difference between the ATP levels observed at pH 7 and 6 increased from 2-fold in exponential phase to about 7- to 8-fold in the stationary phase (Table 3).

**Table 3:** **Intracellular ATP, NADH and NAD**^**+ **^**concentrations, and NADH/NAD**^**+ **^**ratio**

	**pH 7**		**pH 6**	
	**N**_**2 **_**sparging ( *Case I *)**	**No sparging ( *Case II *)**	**N**_**2 **_**sparging ( *Case III *)**	**No sparging ( *Case IV *)**
ATP (μmoles/g biomass)				
Exponential phase	0.097 ± 0.005	0.117 ± 0.003	0.046 ± 0.003	0.043 ± 0.003
Stationary phase	0.254 ± 0.025	0.199 ± 0.017	0.029 ± 0.002	0.030 ± 0.002
NADH (μmoles/g biomass)				
Exponential phase	0.019 ± 0.004	0.021 ± 0.005	0.007 ± 0.002	0.011 ± 0.002
Stationary phase	0.015 ± 0.003	0.025 ± 0.003	0.010 ± 0.002	0.015 ± 0.002
NAD^+^ (μmoles/g biomass)				
Exponential phase	0.239 ± 0.011	0.335 ± 0.032	0.231 ± 0.002	0.271 ± 0.001
Stationary phase	0.201 ± 0.015	0.205 ± 0.038	0.005 ± 0.001	0.010 ± 0.001
NADH/NAD^+^ (mol/mol)				
Exponential phase	0.08 ± 0.02	0.06 ± 0.02	0.03 ± 0.01	0.04 ± 0.01
Stationary phase	0.07 ± 0.01	0.13 ± 0.01	1.77 ± 0.23	1.56 ± 0.39

In the exponential phase the NADH/NAD^+^ ratio measured with and without N_2_ sparging resulted to be similar at both pH levels (Table 3). However, at pH 7 the NADH/NAD^+^ ratio increased by 2-fold (from 0.06 to 0.13) during the stationary phase when *P*_H2_ reached about 56 kPa (*Case II*), while it remained unaffected when *P*_H2_ was kept low by N_2_ sparging (*Case I*). Surprisingly, a dramatic increase of the NADH/NAD^+^ ratio was observed during the stationary phase at pH 6 (*Case III* and *IV*). This was caused by an expected depletion of NAD^+^ pool rather than an increase in intracellular NADH concentration (Table 3).

### Metabolic flux analysis under different pH and *P*_H2_

**Figure 2:**
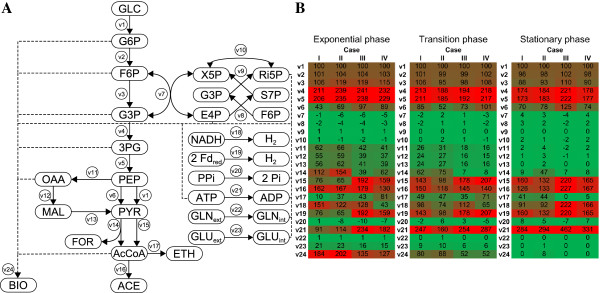
**Metabolic flux distribution of *C. celer *during glucose fermentation at different stages of growth.** Reconstructed metabolic network of central carbon metabolism **(A)** and heatmap of metabolic fluxes normalized with respect to glucose uptake of 100 mmol **(B)**. Stoichiometry for each reaction is defined in Table S1 (Additional file 1). Abbreviations: 3PG, 3-Phosphoglycerate; AcCoA, Acetyl-CoA; ACE, Acetate; ADP, Adenosine diphosphate; ATP, Adenosine triphosphate; BIO, Biomass; E4P, Erythrose-4-phosphate; ETH, Ethanol; F6P, Fructose-6-phosphate; Fd_red_, Reduced ferredoxin; FOR, Formate; G3P, Glyceraldehyde-3-phosphate; G6P, Glucose-6-phosphate; GLC, Glucose; GLN, Glutamine; GLU, Glutamate; H_2_, Hydrogen; MAL, Malate; NADH, Reduced nicotinamide adenine dinucleotide; OAA, Oxaloacetate; Pi, Orthophosphate; PPi, Pyrophosphate; PEP, Phosphoenolpyruvate; PYR, Pyruvate; Ri5P, Ribose-5-phosphate; S7P, Sedoheptulose-7-phosphate; X5P, Xylulose-5-phosphate.

**Figure 3:**
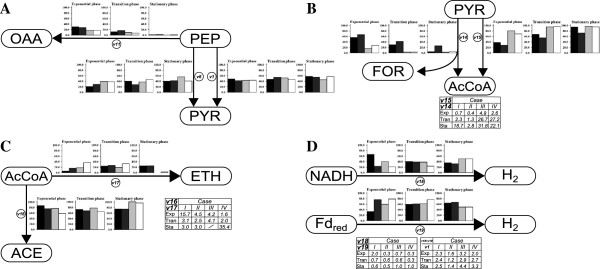
**Node analysis of metabolic fluxes in *C. celer *at different stages of growth.** Fluxes at the phosphoenolpyruvate node **(A)**, pyruvate node **(B)**, acetyl-CoA node **(C)** and H_2_ flux **(D)** at pH 7 with N_2_ sparging (*Case I*, *black*) and without N_2_ sparging (*Case II*, *dark grey*), and at pH 6 with N_2_ sparging (*Case III*, *light grey*) and without N_2_ sparging (*Case IV*, *white*). The values represent the percentage of the flux considering 100 as the incoming flux at the node.

To investigate more in detail the effect of pH and N_2_ sparging on the fermentative metabolism of *C. celer*, metabolic flux analysis (MFA) was carried out for *Case I* to *IV* at different growth phases (Figure 2). A node analysis was performed to estimate the distribution of the fluxes at key metabolic branch points (Figure 3). In the proposed metabolic network of *C. celer* (Figure 2A; Additional file 1: Table S1), phosphoenolpyruvate (PEP) is the first important intermediate being potentially used by three reactions (*v1*, *v6*, *v11*) (Figure 3A). Generally, most of the PEP was converted to pyruvate by reaction *v1* and *v6* especially as the fermentation proceeded. In the exponential phase only a fraction of the PEP was converted to oxaloacetate (*v11*) and almost none in the stationary phase. The distribution at this metabolic node during exponential phase was minimally affected by the difference in *P*_H2_ at both pH. On the other hand, the conversion of PEP to oxaloacetate (*v11*) was affected by pH. In fact, at pH 7 (*Case I* and *II*) the PEP directed to the “malate shunt” (*v11*-*v13*) in the exponential phase was almost 2-fold higher than at pH 6 (*Case III* and *IV*). Given the higher growth rate observed at pH 7 (Figure 1A, Table 2), a higher flux through the “malate shunt” was not surprising since it served as the only source of NADPH for biomass synthesis.

**Figure 4:**
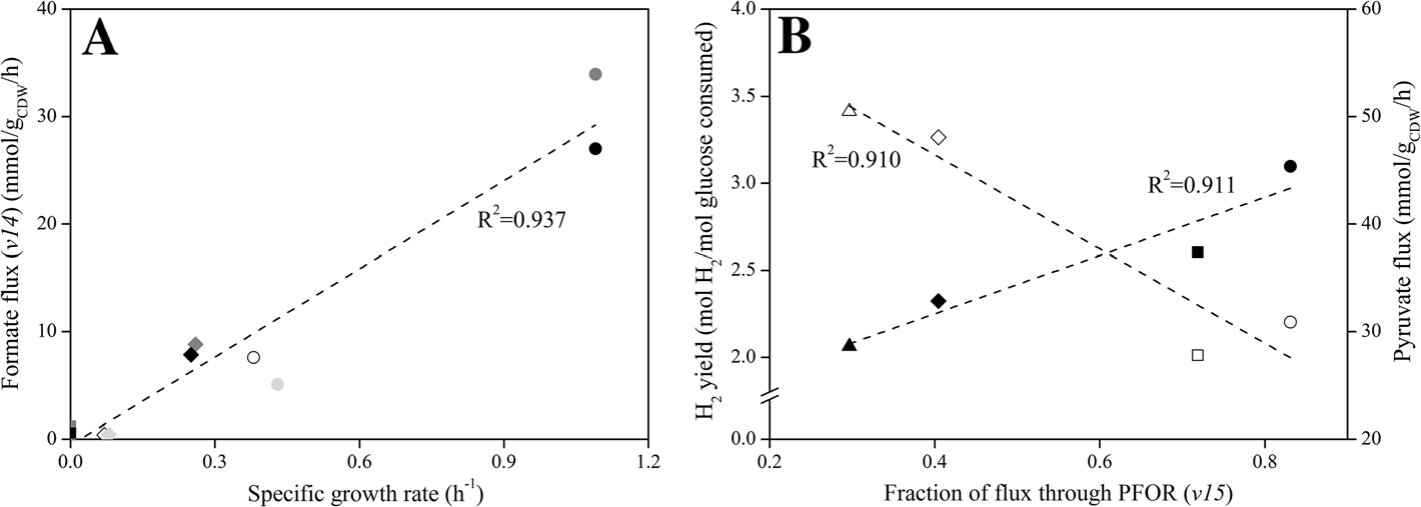
**Correlation between distribution of fluxes at the pyruvate node and growth rate, H**_**2 **_**yield and pyruvate flux.** Correlation between formate flux (*v14*) and growth rate **(A)** in exponential phase (*circles*), transition phase (*diamonds*) and stationary phase (*squares*) at pH 7 with N_2_ sparging (*Case I*, *black*) and without N_2_ sparging (*Case II*, *dark grey*), and at pH 6 with N_2_ sparging (*Case III*, *light grey*) and without N_2_ sparging (*Case IV*, *white*). Correlation between fraction of flux through PFOR during exponential phase and pyruvate flux during exponential phase (*open symbols*), and overall H_2_ yield (*filled symbols*) **(B)** at pH 7 with N_2_ sparging (*Case I*, *diamonds*) and without N_2_ sparging (*Case II*, *triangles*), and at pH 6 with N_2_ sparging (*Case III*, *circles*) and without N_2_ sparging (*Case IV*, *squares*).

**Figure 5:**
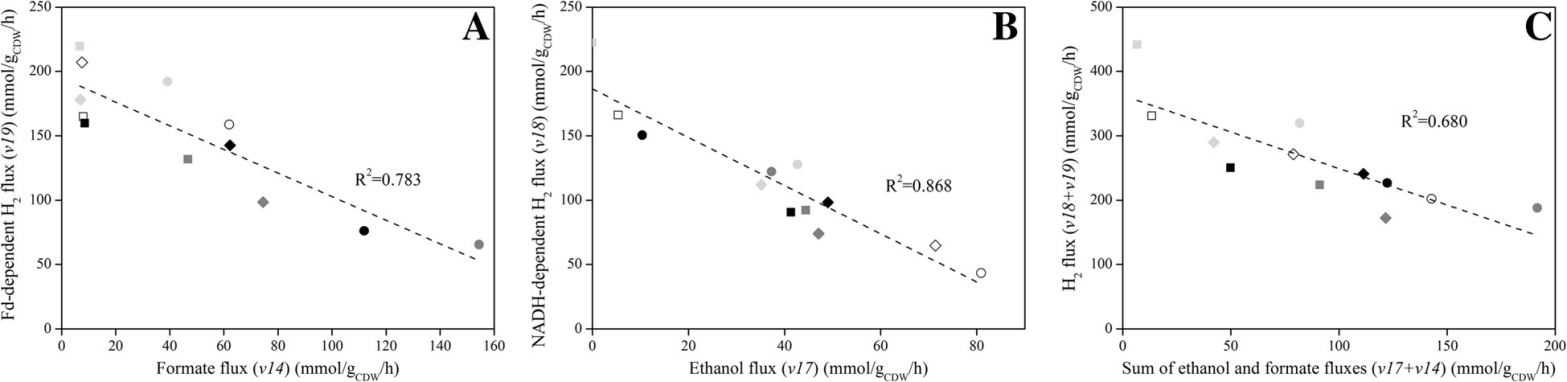
**Correlation between formate and ethanol fluxes, and H**_**2 **_**fluxes.** Correlation between formate flux (*v14*) and Fd-dependent H_2_ flux (*v19*) **(A)**, between ethanol flux (*v17*) and NADH-dependent H_2_ flux (*v18*) **(B)**, and between the sum of formate and ethanol fluxes (*v14*+*v17*) and overall H_2_ flux (*v18*+*v19*) **(C)** in exponential phase (*circles*), transition phase (*diamonds*) and stationary phase (*squares*) at pH 7 with N_2_ sparging (*Case I*, *black*) and without N_2_ sparging (*Case II*, *dark grey*), and at pH 6 with N_2_ sparging (*Case III*, *light grey*) and without N_2_ sparging (*Case IV*, *white*).

The distribution of the flux at the pyruvate node determines the size of the reduced ferredoxin (Fd_red_) available for H_2_ production since only conversion of pyruvate to acetyl-CoA by pyruvate:ferredoxin oxidoreductase (PFOR) generates reducing equivalents. Both pH and *P*_H2_, as well as the growth phase, influenced the fluxes at this node (Figure 3B). When H_2_ was stripped from the reactor, the *v15*/*v14* ratio in the exponential phase almost doubled both at pH 7 (*Case I*) and pH 6 (*Case III*) suggesting that lower *P*_H2_ directs the flux through PFOR. A more dramatic effect on flux distribution at this node was exerted by the culture pH. In the exponential phase neutral pH favored acetyl-CoA formation through pyruvate formate lyase (PFL) (*v15*/*v14* < 1 in *Case I* and *II*), whereas at acidic pH pyruvate was mainly directed through PFOR (*v15*/*v14* > 1 in *Case III* and *IV*). Although decreased in the later stage of growth, a significant formate flux was measured at pH 7 when sparging was not applied (*Case II*), whereas it almost ceased in all other conditions (*Case I*, *III* and *IV*). In general, a direct correlation was observed between formate flux (*v14*) and the growth rate (Figure 4A). Routing the carbon and electron flow through PFL has negative implication for H_2_ production since this reaction does not supply Fd_red_. This is confirmed by the negative correlation existing between the formate flux (*v14*) and the Fd-dependent H_2_ flux (*v19*) estimated at different growth phases (Figure 5A). In addition, the importance of the flux distribution at the pyruvate node for H_2_ production in *C. celer* is also highlighted by the correlation between the fraction of the flux through PFOR in the exponential phase and the overall H_2_ yield (Figure 4B).

Acetyl-CoA is another critical intermediate in *C. celer* metabolism. Conversion of acetyl-CoA to acetate (*v16*) generates one extra ATP, while ethanol production is used as an electron sink for the oxidation of NADH (*v17*). Although production of acetate was generally favored, ethanol flux varied depending of the experimental conditions (Figure 3C). Applying N_2_ sparging to prevent H_2_ building up in the reactor promoted a lower ethanol flux in the exponential phase (*Case I* and *III*). In particular, the flux directed to ethanol was 3- and 2-fold lower for *Case I* and *III* respectively, and similarly the *v16*/*v17* ratio was 3- and 2.5-fold higher suggesting that low *P*_H2_ favored acetate over ethanol production in the exponential phase. Ethanol flux in the exponential phase was lower at pH 7 compared to pH 6 at both sparging conditions. However, while at pH 6 (*Case III* and *IV*) the ethanol production completely ceased in the stationary phase, at pH 7 (*Case I* and *II*) it started increasing at the end of the exponential phase resulting in about 25% of the flux to be directed to ethanol regardless of the *P*_H2_ in the reactor. Since conversion of acetyl-CoA to ethanol requires NADH as reduced cofactor, it competes with NADH-dependent H_2_ production for the use of reducing equivalents. Figure 5B supports this scenario showing that in *C. celer* NADH-dependent H_2_ flux (*v18*) estimated at different growth phases was negatively correlated with ethanol flux (*v17*).

Data from MFA was also used to analyze the H_2_ flux and how its two components, the NADH-dependent (*v18*) and the Fd-dependent (*v19*) H_2_ production, contribute to it (Figure 3D). In the exponential phase N_2_ sparging had a great effect on the NADH-dependent H_2_ flux (*v19*) that increased from 23.4 (*Case II*) to 66.5% (*Case I*) of the total H_2_ flux at pH 7 and from 20.9 (*Case IV*) to 40.2% (*Case III*) at pH 6. However, no evident trend can be identified in the distribution of H_2_ flux with respect of the culture pH in this growth phase. In general, with the exception of *Case I*, during the exponential phase Fd-dependent proton reduction (*v19*) was the favorite reaction for H_2_ synthesis. As the fermentation entered in the stationary phase, at pH 7 (Case *I* and *II*) Fd-dependent H_2_ flux (*v19*) was almost twice the NADH-dependent H_2_ flux (*v18*), while at pH 6 (Case *III* and *IV*) the H_2_ flux was equally distributed between the Fd-dependent (*v19*) and the NADH-dependent reaction (*v18*). When comparing the overall H_2_ flux (*v18* + *v19*) normalized by glucose uptake (*v1*) it is clear that low *P*_H2_ achieved by N_2_ sparging (Case *I* and *III*) promoted higher H_2_ production in *C. celer*, whereas when H_2_ was allowed to accumulate in the reactor (Case *II* and *IV*) H_2_ evolution suffered (Figure 3D). Similarly, running the fermentation at acidic pH (Case *III* and *IV*) instead of at neutral pH (Case *I* and *II*) increased the disposal of reducing equivalent through H_2_ production, especially in the stationary phase when fluxes of the competing reactions (*v14* and *v17*) were minimal (Figure 5C).

## Discussion

### Generation and consumption of reducing equivalents in *C. celer*

During glucose catabolism reducing equivalents are generated in the form of NADH or Fd_red_. These cofactors need to be reoxidized allowing the glycolytic flux to proceed. During fermentation this can be achieved through different reactions involving the synthesis of reduced molecules such as H_2_, ethanol, lactate, butyrate and alanine. Specifically, *C. celer* can produce NADH during the oxidation of glyceraldehyde-3-phosphate by an NAD^+^-dependent glyceraldehyde-3-phosphate dehydrogenase (GAPDH, TCEL_00702) and Fd_red_ in the oxidation of pyruvate to acetyl-CoA by pyruvate:ferredoxin oxidoreductases (PFOR, TCEL_02202-02206/TCEL_01566) (Figure 2A; Additional file 1: Table S1). While in *C. celer* glycolysis (Embden-Meyerhof pathway) yields 2 NADH per one glucose metabolized, generation of Fd_red_ is dependent on flux distribution at the pyruvate node. At this node pyruvate is non-oxidatively dissimilated to acetyl-CoA and formate by pyruvate formate lyase (PFL, TCEL_00503) or oxidatively decarboxylated to acetyl-CoA by PFOR. Only the latter generates Fd_red_ and when 100% of the flux goes through PFOR, generation of reducing equivalents is maximized resulting in the formation of 2 NADH and 4 Fd_red_. If all reduced electron carriers were recycled by hydrogenases, the complete oxidation of one molecule of glucose would yield 4 H_2_ molecules. The genome of *C. celer* contains two clusters coding for putative [FeFe]-hydrogenases (TCEL_00581-00584/TCEL_01273-01277) and one cluster coding for a putative [NiFe]-hydrogenase (TCEL_00187-00205) (Figure 2A; Additional file 1: Table S1). Based on similarity of the gene organization within the clusters and the high homology with the cytosolic enzyme complex of *Caldanaerobacter subterraneus* subsp. *tengcongensis *[12], the two [FeFe]-hydrogenases most likely utilize NADH as an electron donor for proton reduction. The [NiFe]-hydrogenase is predicted to be a multimeric Fd-dependent membrane-bound enzyme similar to the MBH complex characterized in *Pyrococcus furiosus *[20]. In this species MBH couples the oxidation of Fd_red_ with energy conservation by forming an ion gradient that can be used to generate ATP via a membrane-bound ATP synthase.

However, recycling reducing equivalent through H_2_ production is not always thermodynamically favorable. At a given temperature the oxidation of reduced cofactors (particularly of NADH) by hydrogenases is a function of the H_2_ concentration [5]. Supersaturation of the aqueous phase with H_2_ can easily occur due to limitation of liquid-to-gas mass transfer rate [14,15]. As a consequence, at high H_2_ concentrations the disposal of accumulated reducing equivalents in the cell is mediated by a metabolic shift towards production of more reduced metabolites such as ethanol, lactate, and alanine. The genome analysis revealed that in *C. celer* the only alternative for NADH oxidation is ethanol production, since no lactate dehydrogenase nor NAD:ferredoxin oxidoreductase (NFOR) were identified (Figure 2A; Additional file 1: Table S1). In addition, although *C. celer* encodes a complete pathway for butyrate synthesis that would consume NADH, no butyrate was detected as end-product in this study. Ethanol production mediated by putative bifunctional acetaldehyde/alcohol dehydrogenase (TCEL_01373) and alcohol dehydrogenase (TCEL_00064) has been shown to increase at high *P*_H2_ validating the hypothesis that this pathway serves as an alternative to hydrogenase for NADH reoxidation [8,9].

As in *C. celer* the only possibility for Fd_red_ oxidation relies on the activity of the ferredoxin-dependent MBH complex, the distribution of carbon and electron flow at the pyruvate nodes dictates the size of the Fd_red_ pool available for H_2_ production. Despite the similar standard Gibbs energy (ΔG^o^´) [19], the reaction catalyzed by PFOR is expected to be less favorable at high H_2_ concentration due to the increased Gibbs energy (ΔG′) of the ferredoxin oxidation by H_2_ production, whereas the thermodynamics of the reaction catalyzed by PFL does not change. Therefore, in *C. celer* the branched pyruvate node can serve as a safety valve relieving the cell from the burden of ferredoxin reoxidation in unfavorable conditions and thus avoiding a decrease in the metabolic flux.

### Effect of culture pH on growth and fermentative metabolism of *C. celer*

In previous studies performed under non-controlled conditions the optimal initial pH for H_2_ accumulation in *C. celer* was observed to be 8.2 at 67°C (approximately 9.0 at room temperature) [8,9] in accordance with the optimal pH for growth [21]. However, in non-pH-controlled conditions the dynamic pH profile caused by the production of organic acids masked the real effect of pH on the metabolism of *C. celer*. In the current study, pH-controlled fermentations using both 5 and 10 g/l of glucose showed that alkaline to neutral pH (8 and 7 at room temperature) clearly favor high growth rates in *C. celer* confirming the alkalitolerant nature of this organism. On the other hand, at moderately acidic pH (6 and 5.5 at room temperature) the *μ*_max_ was reduced by 2 to 3-fold (Table 1, 2) regardless of the *P*_H2_ in the system. However, with 10 g/l of glucose in the medium, growth ceased before complete glucose depletion and the biomass yield was identical for all the conditions (Table 2). This could be caused by nutrient limitation or by inhibiting conditions such as increased osmolality and accumulation of by-products in the culture [13,22]. When *C. celer* grew at high rates in alkaline/neutral conditions, biomass formation was more energy-efficient despite the lower overall ATP recovery from glucose (Table 1, 3). In contrast, at acidic pH the growth rates were reduced and inefficient biomass formation was observed despite the more effective energy recovery. Given the alkalitolerant nature of *C. celer*, it is likely that at suboptimal extracellular pH this organism needs to maintain the intracellular pH within the physiological range. This goal can be achieved by H^+^-ATPase which exports protons at the expense of ATP hydrolysis. The activity of this enzyme is known to increase at low intracellular pH, thus consuming a substantial portion of the intracellular ATP produced via substrate level phosphorylation [23,24]. Based on the intracellular ATP levels measured at different culture pH conditions (Table 3) and the flux of ATP hydrolysis (*v21*) (Figure 2B), it can be hypothesized that in *C. celer* at lower pH non-growth-associated ATP consumption increases most probably to achieve cytoplasmic pH homeostasis.

An analysis of the end-product distribution clearly indicated that *C. celer* exhibited different metabolic patterns depending on the culture pH. Surprisingly, at moderately acidic pH conversion of glucose to H_2_ was more favorable ( > 2 mol H_2_/mol glucose) than at alkaline to neutral pHs ( < 2 mol H_2_/mol glucose) as a consequence of the lower synthesis of other reduced by-products, i.e. formate and ethanol (Tables 1, 2). On the other hand, volumetric H_2_ productivity was directly correlated with *μ*_max_, both increasing as pH was shifted to alkaline pH. Notably, a transition in the metabolic behavior of *C. celer* was observed between pH 7 and 6 (Tables 1, 2). In particular, both the formate yield and the formate flux during exponential phase were more than 2-fold higher at pH 7 regardless of the *P*_H2_ in the reactor (Table 2; Figures 2B, 3B). A widespread tendency among PFL-encoding organisms to accumulate formate during glucose fermentation as culture pH becomes more neutral/alkaline was observed, albeit with different magnitude, both in pure [25-32] and mixed cultures [33,34]. In absence of a formate hydrogen lyase (FHL) in *C. celer* and other strictly anaerobic bacteria the synthesis of formate by PFL competes with generation of H_2 _[19].

Although at this stage the exact mechanism of this metabolic shift triggered by culture pH in *C. celer* is unknown, several hypotheses can be proposed: i) a simultaneous anabolic and catabolic role of PFL, as reported in closely related clostridia [35], would justify the higher flux through PFL observed at elevated growth rates in this (Figure 4A) and previous studies [9] possibly to meet energy and anabolic demands at optimal pH for growth; ii) since pyruvate can trigger its own utilization by PFL participating as allosteric effector in the activation of this enzyme [36,37] and acetyl-CoA can inhibit PFOR [38-40], at elevated growth rates observed at neutral/alkaline pH higher pyruvate and acetyl-CoA fluxes favored the reaction catalyzed by PFL, whereas pyruvate was mainly directed to PFOR under reduced pyruvate and acetyl-CoA fluxes observed at lower growth rates in acidic conditions and in the transition from the exponential to the stationary phase (Figures 2B, 3B, 4B); iii) the activity of PFL is optimal at pH slightly above 7, but reduced under acidic conditions [37,41]; iv) while the ΔG^o^´ of the reaction catalyzed by PFL is not affected by pH, the reoxidation of Fd_red_ via proton reduction necessary to drive the oxidative pyruvate dissimilation by PFOR, becomes less favorable as the pH increases [27]. Overall, given the direct correlation between flux distribution at the pyruvate node during exponential phase and H_2_ yield (Figure 4B), carbon and electron flow should be channeled through PFOR instead of PFL to maximize H_2_ synthesis.

Unlike formate, ethanol production does not seem to be dependent on pH culture. In fact, at 5 g/l of glucose with N_2_ sparging the highest ethanol yield (0.48 mol/mol glucose) was achieved at pH 7 and the lowest (0.20 mol/mol glucose) at pH 6 (Table 1). However, ethanol yields at pH 8 and 5.5 were similar. Moreover, at high *P*_H2_ no difference was observed between pH 7 and 6 (Table 2). This suggests that regulation of carbon flux through the acetyl-CoA branch point could potentially serve as a means of controlling the disposal of reducing equivalents necessary to maintain an internal redox balance.

### Effect of *P*_H2_ on growth and fermentative metabolism of *C. celer*

The results obtained in this study show that under controlled conditions *P*_H2_ does not affect the growth of *C. celer*. Both biomass formation and bioenergetics parameters (Y_X/ATP_ and Y_ATP/S_) were the same regardless the *P*_H2_ (Figure 1A; Table 2). Also growth rate and consequently volumetric H_2_ productivity were not dependent on *P*_H2_ (Table 2). Under given conditions, the growth rate is dependent on the glycolytic flux which, in turn, is a function of the activity of GAPDH. The activity of GAPDH is negatively affected by an increase of the NADH/NAD^+^ ratio [42,43]. During batch cultivations the NADH/NAD^+^ in *C. celer* was low at exponential phase at both pH (Table 3), suggesting a complete NADH reoxidation regardless of the *P*_H2_. As a consequence, the flux through GAPDH (*v4*) was not inhibited by high *P*_H2_ (Figure 2B). Unlike other anaerobic bacteria which employ alternative pathways to H_2_ production for the reoxidation of NADH (e.g. ethanol and lactate production) only in response to high NADH/NAD^+ ^[43,44], in *C. celer* the ethanol production started already in the exponential phase (Figure 1F) preventing the NADH/NAD^+^ to increase and inhibit GAPDH. As a result, in the conditions at which NADH-dependent H_2_ formation is supposed to be inhibited by high *P*_H2_ (*Case II* and *IV*) ethanol yield is higher (Table 2). NADH was maintained at low levels also in the stationary phase (Table 3). At this stage reoxidation of NADH through ethanol synthesis followed a pH-dependent pattern as at pH 7 ethanol flux (*v17*) and accumulation were still significant, while at pH 6 almost ceased (Figures 1F, 3C).

The pyruvate node is another important metabolic point for the management of reducing equivalents. Indeed, at high *P*_H2_ the overall formate yield and its flux (*v14*) in the exponential phase were higher suggesting that the non-oxidative reaction catalyzed by PFL serves as an alternative route for pyruvate dissimilation at high H_2_ concentration. In fact, while acetyl-CoA production by PFOR produces one pair of reducing equivalents (Fd_red_), in the PFL reaction the reducing equivalents remain with the product formate. Thus, regulation of carbon flux at the pyruvate node could potentially serve as an effective means of controlling the disposal of reducing equivalents necessary to maintain an internal redox balance. Additionally, this electron rerouting was pH-dependent since in *Case II* (pH 7 without N_2_ sparging) formate was accumulated also in the stationary phase and 25.9% of the flux at the pyruvate node was directed through PFL, whereas in all the other conditions formate accumulation and flux almost ceased at this stage of growth. Increased formate yield in response to high *P*_H2_ was reported also for *Clostridium thermocellum *[45,46]. Formate production has never been previously proposed as a pathway to control redox balance in the cell, probably due to the scarce occurrence of a similarly branched pyruvate node in other strict anaerobes.

The majority of the flux being directed to acetate production in all conditions (Figures 2B, 3C) seems to suggest that *C. celer* aims for maximization of ATP production to sustain high growth rate at alkaline/neutral pH or mechanisms for intracellular pH homeostasis at acidic pH. According to the metabolic reconstruction (Figure 2A; Additional file 1: Table S1), four combinations of pathways for glucose catabolism are possible in *C. celer* (Eq. (1-4)):

(1) $$\begin{array}{l} \text{Glucose}+4\;H_{2}O\to 2\;{\text{Acetate}}^{‒}{{+2\;\mathrm{HCO}}_{3}}^{‒}\\ \;+4\;H^{+}+4\;H_{2}\left(4\;\mathrm{ATP}\right) \end{array}$$

(2) $$\begin{array}{l} \text{Glucose}+3\;H_{2}O\to 1\;{\text{Acetate}}^{‒}{{+1\;\text{Ethanol}+2\;\mathrm{HCO}}_{3}}^{‒}\\ \;+3\;H^{+}+2\;H_{2}\left(3\;\mathrm{ATP}\right) \end{array}$$

(3) $$\begin{array}{l} \mathrm{Glucose}+2\;H_{2}O\to 2\;{\text{Acetate}}^{‒}+2\;{\text{Formate}}^{‒}\\ \;+4H^{+}+2\;H_{2}\left(4\;\mathrm{ATP}\right) \end{array}$$

(4) $$\begin{array}{l} \text{Glucose}+1\;H_{2}O\to 1\;{\text{Acetate}}^{‒}+1\;\text{Ethanol}\\ \;+2\;{\text{Formate}}^{‒}+3\;H^{+}\left(3\;\mathrm{ATP}\right) \end{array}$$

Eqs. (1-3) direct 100% of the carbon flux through PFOR (*v15*), whereas Eqs. (3-4) direct the flux entirely through PFL (*v14*). Conversion of glucose to acetate by Eq. (1) allows for maximal ATP generation (4 ATP) by completely relying on hydrogenase activity to regenerate reduced cofactors (NADH and Fd_red_). However, at high H_2_ concentration hydrogenases are inhibited and less-energy efficient pathways for disposal of reducing equivalents need to be activated to sustain the catabolic flux. Indeed, at 70°C and 1 M of dissolved H_2_ this reaction is the least thermodynamically favorable (ΔG′_70°C_ = -153.1 kJ), but becomes more favorable as H_2_ concentration decreases (Additional file 2: Figure S1). The metabolic setup of *C. celer* allows for a reaction yielding 3 ATP (Eq. (4)) whose ΔG′_70°C_ is completely independent of the H_2_ concentration (Additional file 2: Figure S1). Conversely, reactions thermodynamically independent of the H_2_ concentration used by other fermentative bacteria for balancing high NADH/NAD^+^ (e.g. ethanol and lactate synthesis) yield only 2 ATP [12,43,44]. Thus Eq. (4) ensures *C. celer* a higher energy-recovery from glucose breakdown and unaltered glycolytic flux even under unfavorable conditions for H_2_ production. In addition, *C. celer* can potentially still obtain 4 ATP through Eq. (3) which is slightly more favorable at high H_2_ concentrations than Eq. (1) (Additional file 2: Figure S1).

As shown by the inverse correlation between ethanol and formate fluxes with the two components of H_2_ flux (Figure 5A, B, C), ethanol and formate production serves as an alternative to H_2_ production for maintaining the redox balance in the cell when hydrogenases are inhibited: ADH assists NADH-dependent hydrogenase to keep low NADH levels, while PFL simultaneously supplies acetyl-CoA and stores reducing equivalents in formate thus regulating the pool of Fd_red_ available for Fd-dependent hydrogenase. Given the stoichiometry of the fermentations and the distribution of the fluxes, *C. celer* utilizes a combination of the putative pathways to balance anabolic and catabolic requirements as well as intracellular redox state depending on the conditions. One exception can, however, be observed in *Case III* (pH 6 with N_2_ sparging) when *C. celer* entered the stationary phase. Under these conditions an equal contribution by the two components of the H_2_ flux and a normalized H_2_ flux close to the theoretical stoichiometric coefficient of 4 were observed (Figure 3D). Since no ethanol and formate production as well as biomass synthesis were detected, the fermentation proceeded according to Eq. (1). As previously observed [9], in *C. celer* the complete reoxidation of both Fd_red_ and NADH through H_2_ production occurred at low pH and *P*_H2_ when glucose was used for non-growth associated cell maintenance.

## Conclusions

Combining experimental results from batch fermentations, with genome analysis, reconstruction of central carbon metabolism and metabolic flux analysis (MFA), this study shed light on glucose catabolism of the thermophilic alkalitolerant bacterium *C. celer*. This organism possesses a flexible fermentative metabolism that allows efficient energy harvesting from substrate even under unfavorable conditions (i.e. low pH and high *P*_H2_). Two innate factors pertaining to culture conditions have been identified to significantly affect the metabolic flux distribution: culture pH and *P*_H2_.

Overall, at alkaline to neutral pH the rate of biomass synthesis of *C. celer* is maximized and the flux at the pyruvate node mainly directed to PFL suggesting a higher activity of this enzyme as well as a possible role in anabolic metabolism. On the other hand, at acidic pH the lower growth rate and the less efficient biomass formation are accompanied with a more efficient energy recovery from the substrate indicating a high cell maintenance requirement possibly to sustain intracellular pH homeostasis. Higher H_2_ yields were associated with fermentation at acidic pH as a consequence of lower synthesis of other reduced by-products such as formate and ethanol. In contrast, *P*_H2_ does not affect growth of *C. celer* on glucose. At high *P*_H2_ the cellular redox state was balanced by rerouting the flow of carbon and electrons to ethanol and formate production allowing unaltered glycolytic flux and growth rate, but resulting in a decreased H_2_ synthesis. Overall, with the optimization of H_2_ production in mind, *C. celer* offers the flexibility of shifting from a high yield-oriented process (at acidic pH with N_2_ sparging) to a high productivity-oriented process (at alkaline pH without N_2_ sparging). In particular, the tolerance exhibited by *C. celer* towards H_2_ build-up in the reactor is attractive because H_2_ production should be achieved preferably without the need for sparging gas to reduce capital costs for the gas-upgrading process [47]. The metabolic control and regulation of the pyruvate node as well as the genetic, metabolic and physiological traits that allow *C. celer* to withstand high *P*_H2_ merit further studies.

## Materials and methods

### Microorganism and medium

*Caloramator celer* strain JW/YL-NZ35, former *Thermobrachium celere* (equivalent to DSMZ 8682 and ATCC 700318), was obtained from the Deutsche Sammlung von Mikroorganismen und Zellkulturen (Braunschweig, Germany). *C. celer* was cultivated in a modified ATCC 2072 medium.

(http://www.lgcstandards-atcc.org/~/media/4866DB6C29C44938B4B62542D2152259.ashx) containing (per liter): KH_2_PO_4_ 0.75 g; Na_2_HPO_4_ · 2H_2_O 1.53 g; KCl 1 g; (NH_4_)_2_SO_4_ 0.5 g; NH_4_Cl 0.5 g; MgCl_2_ · 6H_2_O 0.1 g; CaCl_2_ · 6H_2_O 0.11 g; FeSO_4_ · 7H_2_O 0.2 g; cystein-HCl 0.2 g; resazurin 0.001 g; trace element solution 10 ml; vitamin solution 10 ml; yeast extract 2 g; tryptone 2 g; glucose 5 or 10 g. Stock solutions under nitrogen atmosphere containing glucose, cystein-HCl, MgCl_2_ · 6H_2_O, CaCl_2_ · 6H_2_O, FeSO_4_ · 7H_2_0 and vitamins were sterilized separately and added anaerobically to autoclaved medium at the required concentrations. Routine subcultures and inoculum development were conducted in 250 ml serum bottles containing 50 ml of medium at 67°C.

### Growth conditions

Cultures were grown in a jacketed, 3-liter bioreactor equipped with an ADI 1025 Bio-Console and an ADI 1010 Bio-Controller (Applikon, Schiedam, The Netherlands) at a working volume of 1 l. The initial pH was maintained at 67°C by automatic titration with 4 M NaOH. The temperature was thermostatically kept at 67 ± 1°C and the stirring rate was set to 250 rpm. A condenser with 5°C cooling water was fitted to the bioreactor's headplate. Prior to inoculation, the medium was sparged with N_2_ and supplemented with an anoxic solution of cysteine-HCl at a final concentration of 0.2 g/l to make the medium completely anaerobic along with the other stock solutions. Glucose was used as a primary substrate in all experiments at an initial concentration of 5 or 10 g/l. The medium was inoculated with 50 ml (5% v/v) of a culture in the exponential phase.

The effect of culture pH on the fermentative metabolism of *C. celer* was studied at four different pH values (8, 7, 6 and 5.5) measured at room temperature. In this experiment the bioreactor was constantly sparged with N_2_ at 100 ml/min. Glucose was added as substrate in concentration of 5 g/l. The effect of *P*_H2_ at two pH levels (7 and 6) was investigated by applying two experimental conditions: continuous N_2_ sparging at 100 ml/min for continuous removal of produced H_2_ (*Case I* at pH 7 and *Case III* at pH 6) and in absence of N_2_ sparging with the bioreactor's gas outlet open to allow accumulation of H_2_ in the headspace at atmospheric pressure (*Case II* at pH 7 and *Case IV* at pH 6). In the latter experimental condition the overall gas production was obtained by measuring the volume of displaced NaHCO_3_-saturated solution in a graduated cylinder. In this experiment glucose was added as substrate in concentration of 10 g/l. Gas samples from the headspace for H_2_ and CO_2_ determination and culture samples for monitoring growth, substrate consumption and metabolite formation were regularly withdrawn during growth. Samples for the measurement of intracellular ATP, NADH and NAD^+^ concentrations were collected as described earlier [48] during the exponential and stationary phase of the cultivations supplemented with 10 g/l glucose. All the experimental conditions were performed in biological duplicates and included negative controls omitting glucose.

### Analytical methods

Headspace samples were analyzed for H_2_ and CO_2_ concentration by gas chromatography, using a dual channel CP-4900 Micro-GC (Varian, Middelburg, The Netherlands) as described earlier [49]. The results were analyzed with a Galaxie Chromatography Workstation (v.1.9.3.2). Cell concentrations were determined by measuring the absorbance at 620 nm using a spectrophotometer (Ultrospec 2100 pro, Amesham Biosciences, UK). Cell dry weight (CDW) was determined as described earlier [44]. Glucose, acetate, formate, ethanol and butyrate were analyzed by HPLC (Waters, Milford, USA) on an Aminex HPX-87H ion exchange column (Bio-Rad, Hercules, USA) at 45°C, with 5 mM H_2_SO_4_ (0.6 ml min^-1^) as the mobile phase. The column was equipped with a refractive index detector (RID-6A, Shimadzu, Japan).

### NAD(H) assay

Cell culture samples (1 ml) were collected in the exponential and stationary phase of growth for NADH and NAD^+^ determination. Samples were immediately quenched by transferring them to a microcentrifuge tube containing 1 ml of ice and centrifuged for 1 min at 12,100 *g*. The pellets were immediately frozen and stored at -80°C until further analysis. Intracellular concentrations of NADH and NAD^+^ were determined by a cyclic assay as described earlier [48]. Intracellular levels of NADH and NAD^+^ were expressed per 1 g of CDW.

### Measurement of ATP

Cell culture samples (1 ml) were collected in the exponential and stationary phase of growth for ATP determinations. Samples were collected in screw-cap microcentrifuge tubes containing 1 ml of ice-cold chloroform and immediately frozen into liquid nitrogen. Samples were stored at -80°C until further analysis. Intracellular concentrations of ATP were measured with an ATP Bioluminescence assay kit HSII (Roche Molecular Biochemicals, Germany) as described earlier [48]. Intracellular levels of ATP were expressed per 1 g of CDW.

### Calculations

H_2_ accumulation (mmol H_2_/l) was calculated in two different ways depending on the experimental design. When N_2_ sparging was applied the calculations were based on the flow rate of the influent N_2_ gas and the percentages of H_2_ and CO_2_ in the effluent gas, whereas when N_2_ sparging was not applied the flow rate of the effluent gas was measured by the liquid displacement method using a NaCO_3_-saturated solution to avoid any further CO_2_ solubilization. Molar H_2_ and CO_2_ were calculated using the ideal gas law based on their concentration in the effluent gas. Volumetric H_2_ productivity (*Q*_H2_; mmol H_2_/l/h) was determined from the slope of the straight line obtained by plotting the cumulative H_2_ (mmol H_2_/l) against the time (h) during exponential growth.

Carbon balances were calculated from the total amount of carbon-containing products formed (in C-mol) and the amount of sugar consumed (in C-mol). Electron balances were calculated after multiplying the amount of each metabolite and sugar by the corresponding degree of reduction (in mol electrons per C-mol). The chemical formula of biomass was assumed to be CH_1.8_O_0.5_ N_0.2_. Metabolite yields as well as carbon and electron balances were calculated by subtracting the background metabolite production in control cultivations (i.e. cultivation without the substrate) and the precultures’ carryover metabolites from the results.

The yield of biomass per ATP (Y_X/ATP_; g_CDW_/mol ATP) and the ATP yield (Y_ATP/S_; mol ATP/mol glucose) were calculated based on the Eq. (5) and Eq. (6), respectively.

(5) $$Y_{X/\mathrm{ATP}}=\frac{\left[\text{biomass}\right]}{2*\left[\text{acetate}\right]+1*\left[\text{ethanol}\right]}$$

(6) $$Y_{\mathrm{ATP}/S}=\frac{2*\left[\text{acetate}\right]+1*\left[\text{ethanol}\right]}{\left[\text{glucose}\;\text{consumed}\right]}$$

The calculation assumes that *C. celer* uses a PEP-dependent phosphotransferase system (PTS) for glucose uptake. Specific rates of substrate consumption uptake and metabolite formation (*r*) were calculated according to

(7) $$r=\frac{\mathrm{dC}}{\mathrm{dt}}*X$$

where *r* is the specific production rate (mmol/g_CDW_/h), C is the substrate or metabolite concentration (mM) and X is the biomass (g_CDW_).

### *In silico* model construction and metabolic flux analysis

The genome of *C. celer* strain JW/YL-NZ35 has been recently sequenced and a high quality draft sequence has been annotated as described earlier [18]. The available genomic data was used to reconstruct the central carbon metabolism of *C. celer* and to build a stoichiometric model including glycolytic pathway, pentose phosphate pathway, end product synthesis pathways, transport mechanisms and biomass synthesis. In order to define each reaction and metabolite in the network, genes encoding for putative enzymes involved in the aforementioned pathways were identified by manual curation of the data from the annotated genome by a combination of sequence alignments [50], gene context analysis, database and literature searches. In addition, physiological evidences were used either when association between genes and reactions could not be established or when a reaction was removed from the network despite a corresponding gene being identified in the genome. The final model consisted of 24 reactions and 20 intracellular metabolites (Additional file 1: Table S1).

Metabolic flux analysis (MFA) was employed to calculate the unknown intracellular fluxes at different stages of *C. celer*´s growth (exponential, transition and stationary phase) under the tested conditions. A metabolic matrix was constructed based on the model according to the law of mass conservation and on the pseudo-steady state hypothesis for the intracellular metabolites. Given the overdetermined nature of the system, the mass balance equations for all the metabolites were expressed in matrix form as:

(8) $$V_{c}=-{G_{c}}^{*}\times G_{m}\times V_{m}\;$$

where V_c_ is the calculated flux vector, G_c_^*^ is pseudo-inverse matrix of the calculated reactions, G_m_ is the matrix of the measured reactions and V_m_ the measured flux vector. The matrix of MFA model was solved with MATLAB R2013a (The MathWorks, Inc., Natick, USA).

## Competing interests

The authors declare that they have no competing interests.

## Authors' contributions

AC, SSP and EWJvN designed the study. AC and SSP planned and preformed the batch experiments. AC planned and preformed NAD(H) and ATP quantifications and metabolite analysis. AC and SSP performed metabolic flux analysis. AC wrote the manuscript. SSP, VS, MK and EWJvN participated in manuscript drafting. VS, MK and EWJvN supervised and coordinated the study. All authors have read and approved the manuscript.

## Supplementary Material

Additional file 1: Table S1 Reconstructed genomic model of the central carbon metabolism in *Caloramator celer*. Click here for file

Additional file 2: Figure S1 Effect of the H_2_ concentration on the Gibbs energy change (ΔG´) at 70°C of four predicted reactions involved in glucose fermentation in C. celer. Click here for file
