# Supplementary material for: Assessment of metabolic flux distribution in the thermophilic hydrogen producer Caloramator celer as affected by external pH and hydrogen partial pressure
Source: Microb Cell Fact. 2014 Mar 28;13:48. doi: 10.1186/1475-2859-13-48 (PMC3986597; doi:10.1186/1475-2859-13-48)
Supplement: Additional file 1: Table S1 — Reconstructed genomic model of the central carbon metabolism in Caloramator celer. [file 1475-2859-13-48-S1.docx]

**Additional file 1**

Table S1. **Reconstructed genomic model of the central carbon metabolism in *Caloramator celer*.**

| **Pathway** |  | **E.C.** |  | **Enyzme** |  | **Locus Tag** |  | **Model reaction** |  | **Stoichiometric equation** |
| --- | --- | --- | --- | --- | --- | --- | --- | --- | --- | --- |
|  |  |  |  |  |  |  |  |  |  |  |
|  |  |  |  |  |  |  |  |  |  |  |
| *Embden–Meyerhof* |  |  |  |  |  |  |  |  |  |  |
|  |  | 2.7.3.9 |  | Glucose-specific PTS system |  | TCEL_00590-588 |  | v1 |  | GLU + PEP → G6P + PYR |
|  |  | 2.7.1.69 |  |  |  | TCEL_02105 |  |  |  |  |
|  |  | 5.3.1.9 |  | Glucose-6-P isomerase |  | TCEL_00792 |  | v2 |  | G6P → F6P |
|  |  | 2.7.1.1 |  | 6-phosphofructokinase |  | TCEL_00490 |  | v3 |  | F6P + ATP → 2 GAP |
|  |  | 4.1.2.13 |  | Fructose-1,6-bisphosphate aldolase |  | TCEL_01472 |  |  |  |  |
|  |  | 5.3.1.1 |  | Triosephosphate isomerase |  | TCEL_00700 |  |  |  |  |
|  |  | 1.2.1.12 |  | Glyceraldehyde-3-P dehydrogenase |  | TCEL_00702 |  | v4 |  | GAP + NAD^+^ + ADP + Pi → 3PG + NADH + ATP |
|  |  | 2.7.2.3 |  | Phosphoglycerate kinase |  | TCEL_00701 |  |  |  |  |
|  |  | 5.4.2.1 |  | Phosphoglyceromutase |  | TCEL_00699 |  | v5 |  | 3PG → PEP |
|  |  | 4.2.1.11 |  | Enolase |  | TCEL_00698 |  |  |  |  |
|  |  | 2.7.1.40 |  | Pyruvate kinase |  | TCEL_00489 |  | v6 |  | PEP + ADP → PYR + ATP |
| *Pentose phosphate* |  |  |  |  |  |  |  |  |  |  |
|  |  | 2.2.1.1 |  | Transketolase |  | TCEL_01616-01617 |  | v7 |  | GAP + F6P ↔ E4P + X5P |
|  |  |  |  |  |  |  |  | v8 |  | E4P + F6P ↔ GAP + S7P |
|  |  | 2.2.1.2 |  | Transaldolase |  | TCEL_01175 |  | v9 |  | S7P + GAP ↔ Ri5P + X5P |
|  |  | 5.1.3.1 |  | Ribulose-phosphate 3-epimerase |  | TCEL_01808 |  | v10 |  | Ri5P ↔ X5P |
|  |  | 5.3.1.6 |  | Ribose 5-phosphate isomerase |  | TCEL_01228 |  |  |  |  |
| *Malate shunt* |  |  |  |  |  |  |  |  |  |  |
|  |  | 4.1.1.49 |  | Phosphoenolpyruvate carboxykinase |  | TCEL_01721 |  | v11 |  | PEP + ADP + CO_2_ ↔ OAA + ATP |
|  |  | 1.1.1.37 |  | Malate dehydrogenase |  | TCEL_01684 |  | v12 |  | OAA + NADH ↔ MAL + NAD^+^ |
|  |  | 1.1.1.40 |  | Malic enzyme |  | TCEL_02077 |  | v13 |  | MAL + NADP^+^ ↔ PYR + NADPH + CO_2_ |
| *Fermentation products* |  |  |  |  |  |  |  |  |  |  |
|  |  | 2.3.1.54 |  | Pyruvate formate lyase |  | TCEL_00503 |  | v14 |  | PYR + CoA → FOR + AcCoA |
|  |  | 1.2.7.1 |  | Pyruvate:ferredoxin oxidoreductase |  | TCEL_02202-02206 |  | v15 |  | PYR + 2 Fd_ox_ + CoA → AcCoA + 2 Fd_red_ + CO_2_ |
|  |  |  |  |  |  | TCEL_01566 |  |  |  |  |
|  |  | 2.3.1.8 |  | Phospho-transacetylase |  | TCEL_01822 |  | v16 |  | Pi + ADP + AcCoA → ACE + ATP + CoA |
|  |  | 2.7.2.1 |  | Acetate kinase |  | TCEL_01823 |  |  |  |  |
|  |  | 1.2.1.10 |  | Aldehyde dehydrogenase |  | TCEL_01373 |  | v17 |  | AcCoA + 2 NADH → ETH + 2NAD^+^ |
|  |  | 1.1.1.1 |  | Alcohol dehydrogenase |  | TCEL_01373 |  |  |  |  |
|  |  |  |  |  |  | TCEL_00064 |  |  |  |  |
| *Hydrogen* |  |  |  |  |  |  |  |  |  |  |
|  |  |  |  | NADH-dependent hydrogenase |  | TCEL_00581-00584 |  | v18 |  | NADH + H^+^ ↔ H_2_ + NAD^+^ |
|  |  |  |  |  |  | TCEL_01273-01277 |  |  |  |  |
|  |  |  |  | Ferredoxin-dependent hydrogenase |  | TCEL_00187-00205 |  | v19 |  | 2 H^+^ + 2 Fd_red_ ↔ 2 H_2_ + 2 Fd_ox_ |
|  |  |  |  |  |  |  |  |  |  |  |
|  |  |  |  |  |  |  |  |  |  |  |
| *Generalized energy utilization and others* |  |  |  |  |  |  |  |  |  |  |
|  |  |  |  |  |  |  |  |  |  |  |
|  |  | 3.6.1.1 |  | Inorganic pyrophosphatase |  | TCEL_00208 |  | v20 |  | PPi + H_2_0 → 2 Pi |
|  |  |  |  | V-type pyrophosphatases |  | TCEL_00696 |  |  |  |  |
|  |  | 3.6.3.14 |  | F_0_F_1_-ATP synthase |  | TCEL_01235-01242 |  | v21 |  | ATP + H_2_0 → ADP + Pi |
|  |  |  |  | Amino acid ABC transporter |  | TCEL_02101-02105 |  | v22 |  | ATP + GLN_ext_ + H_2_O ↔ GLN_int_ + ADP + Pi |
|  |  |  |  |  |  | TCEL_00690-00692 |  | v23 |  | ATP + GLU_ext_ + H_2_O ↔ GLU_int_ + ADP + Pi |
| *Biomass* |  |  |  |  |  |  |  |  |  |  |
|  |  |  |  |  |  |  |  | v24 |  | 3.515•10^−2^•3PG + 8.807•10^−2^•AcCoA + 8.483•10^−3^•E4P + 1.666•10^−3^•F6P + 3.031•10^−3^•G3P + 4.817•10^−3^•G6P + 6.009•10^−3^•GLN + 1.161•10^−1^•GLU + 4.199•10^−2^•OAA + 1.219•10^−2^•PEP + 6.657•10^−2^•PYR + 2.109•10^−2^•Ri5P + 1.405•ATP + 3.061•10^−1^•NADPH → BIO + 8.335•10^−2^•NADH + 1.405•Pi |

Abbreviations: 3PG, 3-Phosphoglycerate; AcCoA, Acetyl-CoA; ACE, Acetate; ADP, Adenosine diphosphate; ATP, Adenosine triphosphate; BIO, Biomass; CO_2_, Carbon dioxide; CoA, Coenzyme A; E4P, Erythrose-4-phosphate; ETH, Ethanol; F6P, Fructose-6-phosphate; Fd_ox_, Oxidized ferredoxin; Fd_red_, Reduced ferredoxin; FOR, Formate; G3P, Glyceraldehyde-3-phosphate; G6P, Glucose-6-phosphate; GLC, Glucose; GLN, Glutamine; GLU, Glutamate; H_2_, Hydrogen; H_2_O, Water; MAL, Malate; NAD^+^, Oxidized nicotinamide adenine dinucleotide; NADH, Reduced nicotinamide adenine dinucleotide; NADP^+^, Oxidized nicotinamide adenine dinucleotide phosphate; NADPH, Reduced nicotinamide adenine dinucleotide phosphate; OAA, Oxaloacetate; Pi, Orthophosphate; PPi,Pyrophosphate; PEP, Phosphoenolpyruvate; PYR, Pyruvate; Ri5P, Ribose-5-phosphate; S7P, Sedoheptulose-7-phosphate; X5P, Xylulose-5-phosphate.
