# Supplementary material for: Assessment of metabolic flux distribution in the thermophilic hydrogen producer Caloramator celer as affected by external pH and hydrogen partial pressure
Source: Microb Cell Fact. 2014 Mar 28;13:48. doi: 10.1186/1475-2859-13-48 (PMC3986597; doi:10.1186/1475-2859-13-48)
Supplement: Additional file 2: Figure S1 — Effect of the H2 concentration on the Gibbs energy change (ΔG´) at 70°C of four predicted reactions involved in glucose fermentation in C. celer. [file 1475-2859-13-48-S2.docx]

**Additional File 2**

Figure S1. **Effect of the H_2_ concentration on the Gibbs energy change (ΔG´) at 70°C of four predicted reactions involved in glucose fermentation in *C. celer*.** Eq. (1) (*filled diamonds*), Eq. (2) (*filled circles*), Eq. (3) (*open circles*) and Eq. (4) (*open diamonds*). Calculations were modified from Bielen *et al*, 2013 [1]


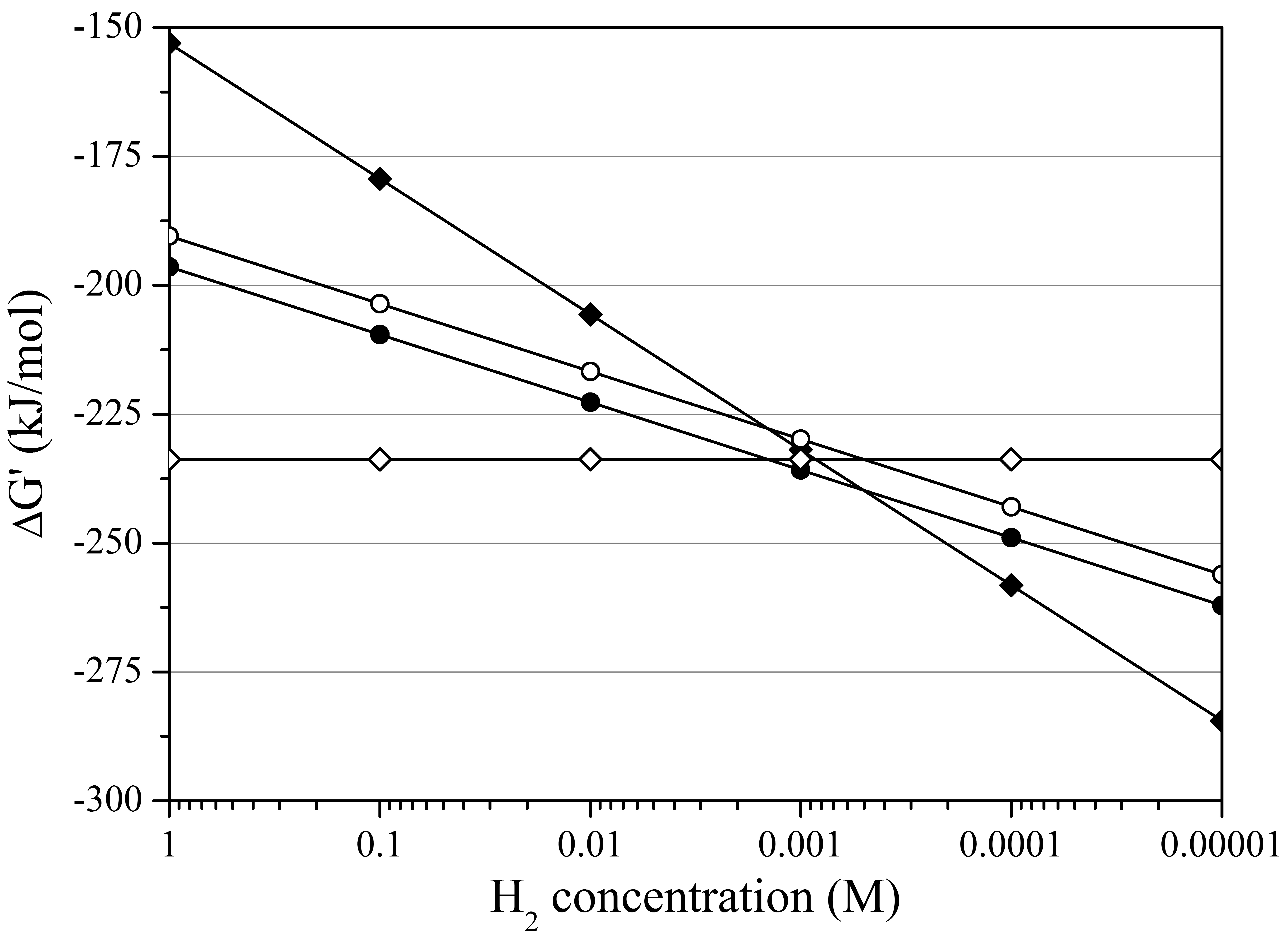


**References**

1. Bielen AAM, Verhaart MRA, van der Oost J, Kengen SWM: **Biohydrogen Production by the Thermophilic Bacterium *Caldicellulosiruptor saccharolyticus*: Current Status and Perspectives.** *Life* 2013, **3**:52-85.
